# Combined morphologic and immunohistochemical assessment of KIM-1-positive proximal tubular vacuolation in one-hour kidney allograft biopsies

**DOI:** 10.1186/s12882-026-05088-5

**Published:** 2026-06-05

**Authors:** Keishu Kawanishi, Mayu Shimokawa, Dedong Kang, Masaki Baba, Yojiro Kato, Osamu Yoshitake, Fumihiko Koiwa, Kazuho Honda, Kunio Kawanishi

**Affiliations:** 1 Department of Anatomy, Showa Medical University School of Medicine, Hatanodai, Shinagawa-ku, Tokyo, Japan; 2 Internal Medicine (Nephrology), Showa Medical University Fujigaoka Hospital, Yokohama, Kanagawa Japan; 3 Department of Diagnostic Pathology, Institute of Medicine, University of Tsukuba, Ibaraki, Japan; 4 Kidney Transplant Center, Showa Medical University Hospital, Shinagawa-ku, Tokyo, Japan; 5 Department of Pathology and Laboratory Medicine, Showa Medical University School of Medicine, Hatanodai, Shinagawa-ku, Tokyo, Japan; 6 Department of Experimental Pathology, Institute of Medicine, University of Tsukuba, Ibaraki, Japan

**Keywords:** Acute tubular injury, Cold ischemia time, Hypoxia-inducible factor-1 alpha (HIF-1α), Ischemia–reperfusion injury, Kidney injury molecule-1 (KIM-1), Kidney transplantation, Proximal tubular vacuolation, Ultra-early epithelial stress

## Abstract

**Background:**

Early kidney allograft injury immediately after reperfusion remains incompletely characterized, particularly before overt hypoxic or transcriptional responses develop. Kidney injury molecule-1 (KIM-1) is rapidly induced in injured proximal tubular epithelial cells; however, the significance of morphological changes within KIM-1–positive epithelium during the ultra-early post-transplant period remains unclear. This study aimed to characterize early epithelial instability in one-hour kidney allograft biopsies and assess associations with ischemic stress and early graft function.

**Methods:**

We retrospectively analyzed one-hour post-reperfusion kidney allograft biopsies from 51 adult recipients who underwent living-donor kidney transplantation. KIM-1 immunohistochemistry was used to identify injured proximal tubules in morphologically non-atrophic cortical regions. Detectable staining in a subset of proximal tubular epithelial cells was used to identify evaluable tubules. Within these KIM-1–positive tubules, epithelial changes including cytoplasmic vacuolation, brush border loss, and focal epithelial detachment were semi-quantitatively graded as a KIM-1–associated vacuolation score (range 0–3), designed to reflect epithelial structural instability rather than the extent of KIM-1 expression. Digital image analysis quantified KIM-1–positive tubular area and hypoxia-inducible factor-1α (HIF-1α) nuclear staining. Associations with cold ischemia time and early post-transplant estimated glomerular filtration rate were assessed using correlation analyses and multivariable linear regression models.

**Results:**

KIM-1–positive proximal tubules were identified in morphologically preserved cortical regions one hour after reperfusion. The KIM-1–associated vacuolation score showed a weak positive trend with cold ischemia time, whereas KIM-1–positive tubular area did not. Higher vacuolation scores tended to be associated with lower estimated glomerular filtration rate at one week, although the association was not statistically significant. In contrast, HIF-1α nuclear accumulation was minimal at this ultra-early time point and showed no association with the vacuolation score, suggesting a dissociation between early epithelial instability and hypoxia-driven transcriptional responses.

**Conclusions:**

Combined assessment of KIM-1 expression and proximal tubular vacuolation in one-hour reperfusion biopsies captured morphologic features occurring during the ultra-early phase after kidney transplantation, when overt HIF-1α activation was minimal. Although no significant associations with early graft function were observed, this proof-of-concept approach may provide a framework for future studies investigating ultra-early epithelial stress responses in kidney transplantation.

**Supplementary Information:**

The online version contains supplementary material available at 10.1186/s12882-026-05088-5.

## Background

Kidney transplantation remains the optimal treatment for end-stage renal disease; however, early allograft dysfunction continues to negatively affect short- and long-term outcomes [1]. Ischemia–reperfusion injury is central to the development of early tubular epithelial damage, and its severity at the moment of reperfusion strongly influences delayed graft function and the trajectory of early graft recovery [2, 3]. Identifying biomarkers capable of capturing these ultra-early epithelial responses is therefore of substantial clinical importance.

Kidney injury molecule-1 (KIM-1) is a proximal tubular transmembrane glycoprotein that is rapidly induced during acute epithelial injury [4, 5]. It reflects early membrane perturbation, cytoskeletal remodeling, and brush border loss, and has been widely studied as a sensitive indicator of ischemic tubular damage [5, 6]. While most investigations have focused on urinary KIM-1 or its expression several hours to days after injury, far less is known about KIM-1 dynamics within the first hour following renal allograft reperfusion. This immediate period represents a distinct biological state in which epithelial cells transition abruptly from cold preservation to warm, oxygenated circulation, undergoing metabolic reactivation, mitochondrial stress, and early membrane injury before overt necrotic changes appear [6, 7].

Baseline biopsies obtained one hour after graft reperfusion provide a unique opportunity to characterize these earliest events. Among the morphological alterations observed in this phase, proximal tubular vacuolation is frequently encountered and may reflect endocytic overload or sublethal membrane injury [6, 8]. However, the relationship between KIM-1 expression and vacuolation in this ultra-early window has not been systematically examined, nor has the combined morphologic and immunohistochemical significance of these findings during the early reperfusion phase been systematically evaluated.

Cold ischemia time (CIT) is conventionally regarded as a major determinant of ischemia–reperfusion injury, with longer preservation typically associated with more severe tubular damage [9]. Yet recent data suggest that the biological response to cold preservation may be influenced by the depth of metabolic suppression achieved during hypothermic storage, which can reduce ATP consumption and reactive oxygen species generation and thereby attenuate abrupt reperfusion-related stress [6, 10, 11]. In contrast, kidneys with shorter preservation times may retain higher metabolic activity at reperfusion and thus experience more pronounced early epithelial injury. The dynamics of these interactions at one hour after reperfusion remain poorly understood.

In addition, hypoxia-inducible factor-1α (HIF-1α), which stabilizes during later phases of reperfusion and contributes to adaptive or protective responses, is often absent in the ultra-early period [12]. The lack of HIF-1α expression in one-hour biopsies may indicate that the morpho-molecular state captured by KIM-1 and vacuolation precedes the onset of classical hypoxic signaling.

Taken together, these observations highlight an important gap in our understanding of the interplay between cold ischemia duration, early epithelial morphology, and immediate molecular responses in the transplanted kidney. Clarifying how KIM-1 expression and tubular vacuolation behave within the first hour after reperfusion, and how these features relate to CIT and early postoperative recovery, may provide insight into ultra-early epithelial stress responses following kidney transplantation.

The present study therefore aimed to evaluate proximal tubular KIM-1 expression and associated vacuolation in one-hour baseline biopsies after renal transplantation, to determine their relationship with cold ischemia time, and to assess their potential value as early markers of graft recovery.

## Methods

### Ethics approval

The study protocol was approved by the Institutional Review Board of Showa Medical University Hospital, Tokyo, Japan (Approval No. 21–230-A).

Details regarding ethical considerations, including consent procedures, are provided in the Declarations section. A flow diagram illustrating patient eligibility, exclusion, and inclusion in the final analysis is presented in Supplementary Figure 1.

### Immunohistochemistry

Immunohistochemistry was performed on formalin-fixed, paraffin-embedded tissue sections using an automated staining system (HISTOSTAINER 48A; Nichirei Biosciences, Tokyo, Japan). Antigen retrieval was carried out with EnVision FLEX Target Retrieval Solution (Agilent Technologies, Santa Clara, CA, USA) at 97 °C for 20 min, using high-pH buffer for HIF-1α and low-pH buffer for KIM-1. HIF-1α was detected using a rabbit monoclonal anti-human HIF-1α antibody (Cell Signaling Technology, Danvers, MA, USA; clone E1V6A; 1:100), followed by blocking with an animal-free blocking solution and signal detection using POLYVIEW® PLUS HRP Reagent (Enzo Life Sciences, Farmingdale, NY, USA) with 3,3′-diaminobenzidine (DAB) as the chromogen. KIM-1 was detected using a goat polyclonal anti-human TIM-1/KIM-1/HAVCR antibody (Bio-Techne, Minneapolis, MN, USA; 1:200). After blocking with normal horse serum, signal detection was performed using ImmPRESS-HRP Horse Anti-Goat IgG Polymer (Vector Laboratories, Newark, CA, USA) and DAB. All sections were counterstained with hematoxylin, dehydrated, and mounted. Whole-slide images were acquired using a NanoZoomer S360MD scanner (Hamamatsu Photonics, Hamamatsu, Japan).

### Histological evaluation and KIM-1–associated vacuolation scoring

KIM-1–associated vacuolation was graded on a four-tier scale (score 0–3) defined as follows: Score 0: No detectable KIM-1 staining in proximal tubular epithelial cells, with preserved cytoplasmic architecture and absence of vacuolation. Brush borders and tubular integrity are maintained.

Score 1: Focal or weak KIM-1 positivity limited to a small subset of proximal tubular epithelial cells, accompanied by minimal cytoplasmic vacuolation. Vacuoles are sparse and do not distort tubular architecture.

Score 2: Moderate KIM-1 expression involving a substantial proportion of proximal tubules, associated with clearly identifiable cytoplasmic vacuolation. Vacuoles are increased in number and size, partially displacing the cytoplasm while epithelial continuity is preserved.

Score 3: Strong and diffuse KIM-1 expression in proximal tubular epithelial cells, accompanied by prominent and widespread vacuolation. Vacuoles occupy a large portion of the cytoplasm, and focal epithelial cell detachment or denudation of the tubular basement membrane may be observed, indicating severe epithelial stress at the ultra-early reperfusion phase.

Scoring was performed independently and anonymously by three investigators (Keishu Kawanishi, Dedong Kang, and Kazuho Honda), all of whom were blinded to clinical information, including cold ischemia time and postoperative graft outcomes. For each biopsy, representative cortical fields meeting the predefined inclusion criteria were evaluated, and the predominant score was assigned. Discrepant cases were resolved by consensus review. This score was designed to prioritize epithelial structural instability within KIM-1–positive proximal tubules rather than the extent of KIM-1 positivity itself. Morphological vacuolation was evaluated in conjunction with KIM-1 immunoreactivity because KIM-1 staining facilitated the identification of injured proximal tubular epithelial cells within morphologically preserved cortical regions and helped distinguish true cytoplasmic vacuolation from processing-related artifacts. Therefore, this score was intended to assess vacuolar change specifically within KIM-1–positive injured proximal tubules, rather than vacuolation in all tubules irrespective of injury-marker expression.

### Digital image analysis

Whole-slide images of immunohistochemically stained sections were acquired using a NanoZoomer S60 scanner (Hamamatsu Photonics, Hamamatsu, Japan) and analyzed with QuPath (version 0.6.0) [13]. All slides were scanned at high resolution, and identical analysis pipelines were applied across cases.

To focus on ultra-early tubular epithelial injury, regions of interest (ROIs) were manually defined prior to automated analysis. Glomeruli and overtly atrophic areas—characterized by tubular thinning, luminal collapse, interstitial expansion, or fibrosis (Supplemental Figure 2)—were excluded. After exclusion, two cortical ROIs were generated for each case: (1) a whole cortical tubular ROI encompassing all remaining cortical tubules, and (2) a medullary ray–restricted ROI defined by radially oriented tubular structures extending from the corticomedullary junction. All ROIs were reviewed by experienced observers to ensure anatomical consistency. Within each ROI, a machine-learning–based pixel classifier was used to identify tubular epithelium, which served as the denominator for analysis. KIM-1 expression was detected using DAB chromogen and quantified by color deconvolution–based thresholding applied uniformly across slides. KIM-1–positive tubular area was calculated as the percentage of DAB-positive area relative to total tubular epithelial area, separately for whole cortical and medullary ray ROIs. HIF-1α expression was assessed within the whole cortical ROI using automated nuclear detection. Given the sparse nuclear staining at one hour after reperfusion, HIF-1α activation was summarized using a simplified H-score–based metric, calculated as the product of the proportion of positive nuclei and mean staining intensity. Cases with insufficient staining quality were excluded from HIF-1α quantitative analysis. QuPath-derived quantitative metrics were analyzed alongside the semi-quantitative KIM-1–associated vacuolation score, which was designed to capture qualitative epithelial instability within KIM-1–positive proximal tubules and was analyzed independently of the extent of KIM-1–positive area.

**Fig. 2:**
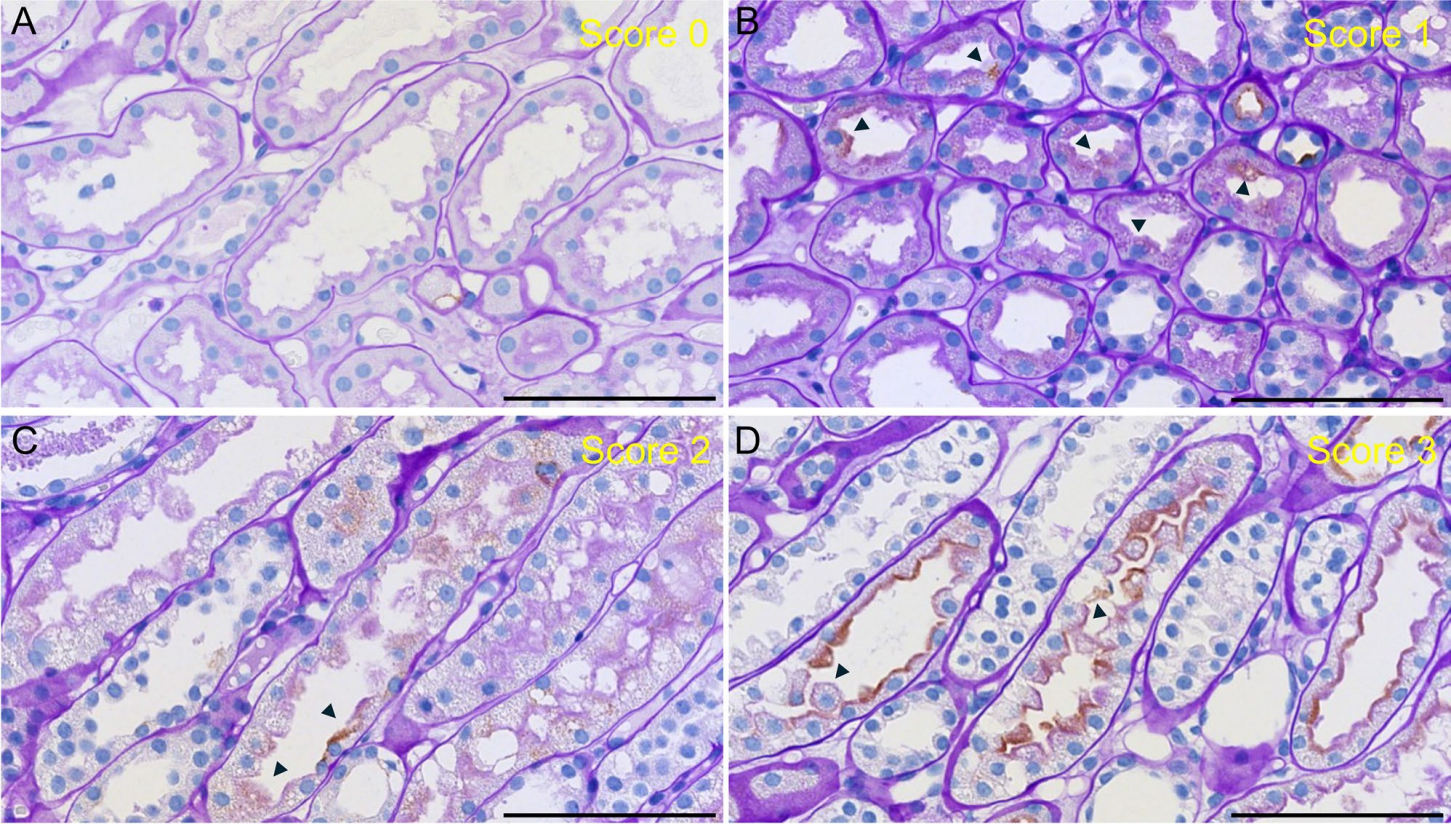
KIM-1–associated proximal tubular vacuolation scoring in one-hour kidney allograft biopsies. Representative photomicrographs illustrate the semi-quantitative scoring system for ultra-early proximal tubular injury based on KIM-1 expression and cytoplasmic vacuolation in one-hour reperfusion biopsies. (**A**) score 0: proximal tubules show preserved epithelial morphology with intact cytoplasm and no detectable KIM-1 immunoreactivity; vacuolation is absent. (**B**) score 1: focal and weak KIM-1 positivity in a limited number of proximal tubular epithelial cells (arrowheads), accompanied by minimal cytoplasmic vacuolation without architectural distortion. (**C**) score 2: moderate KIM-1 expression in multiple proximal tubules with clearly visible cytoplasmic vacuolation (arrowheads); epithelial continuity is preserved. (**D**) score 3: strong and diffuse KIM-1 immunoreactivity associated with prominent and extensive vacuolation (arrowheads); vacuoles occupy a large portion of the cytoplasm, and focal epithelial cell detachment may be present, indicating severe ultra-early epithelial stress following reperfusion. Scale bars: 100 μm

### Statistical analysis

All statistical analyses were performed using R software (version 4.5.0). Continuous variables are presented as mean ± standard deviation or median with interquartile range, as appropriate. Associations between the KIM-1–associated vacuolation score and continuous variables were assessed using Pearson or Spearman correlation analyses, depending on data distribution, and visualized using linear regression with 95% confidence intervals.

Renal function at 1 week after transplantation was assessed using estimated glomerular filtration rate (eGFR) to allow exploratory comparison of early graft function across recipients. We acknowledge that creatinine kinetics may not be fully stabilized at this early post-transplant time point; therefore, eGFR was not intended as a definitive prognostic marker but rather as a reference indicator of early functional recovery. Serum creatinine values at 1 week are additionally presented in the table to provide complementary clinical context. The model included the KIM-1–associated vacuolation score, KIM-1–positive tubular area, cold ischemia time, and pre-transplant tacrolimus concentration. All continuous variables were standardized prior to analysis, and results are presented as standardized β coefficients with 95% confidence intervals. Group-wise comparisons were performed using Kruskal–Wallis tests. Analyses were conducted using complete-case analysis. All tests were two-sided, with *p* < 0.05 considered statistically significant. Given the exploratory nature and limited sample size, effect sizes and confidence intervals were emphasized.

## Results

### Identification of evaluable proximal tubules for ultra-early injury assessment in one-hour reperfusion biopsies

**Fig. 1:**
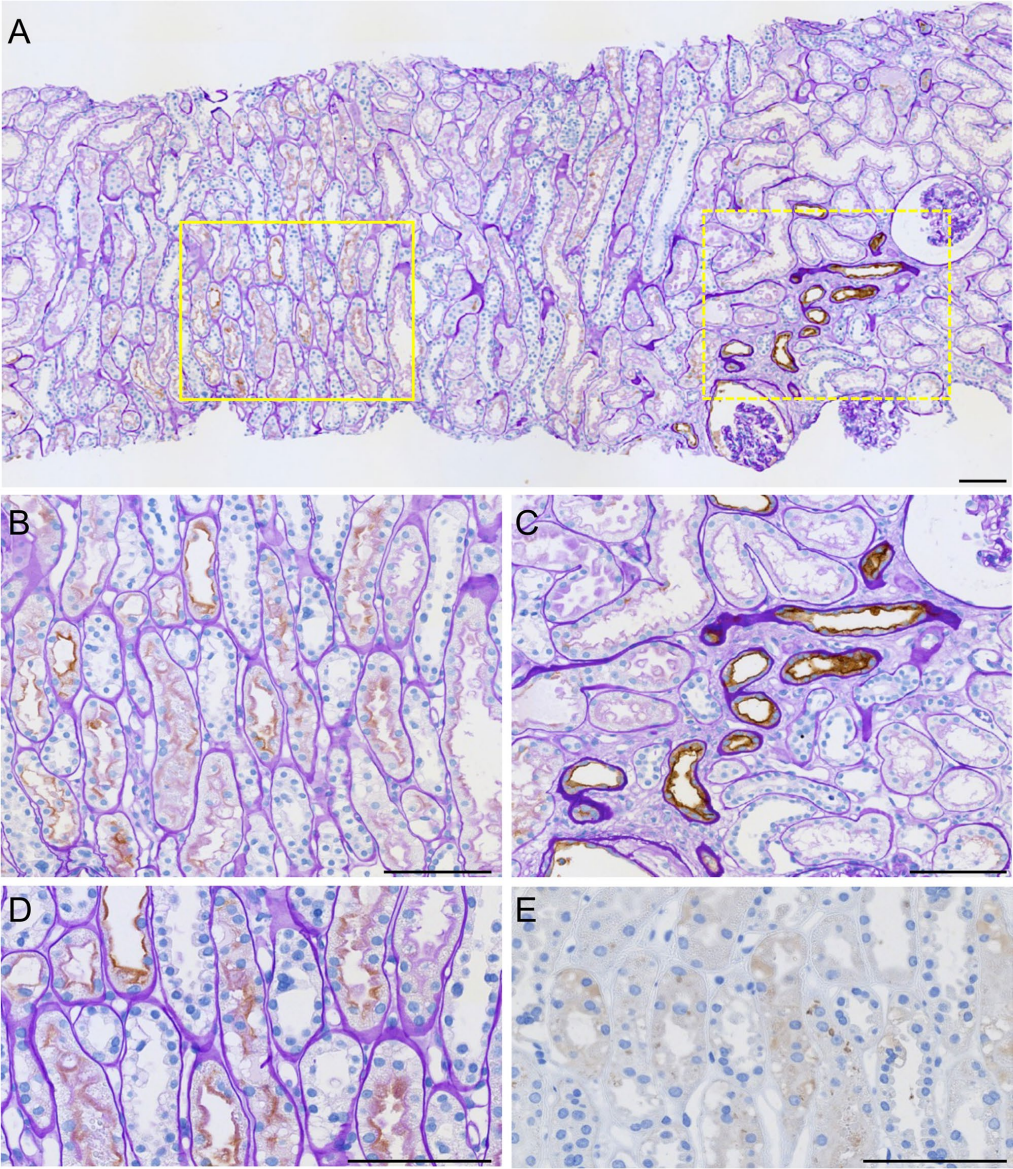
Identification of target proximal tubules for vacuolation assessment using combined KIM-1 immunohistochemistry and PAS staining in one-hour kidney allograft biopsies. (**A**) low-power overview of a one-hour reperfusion biopsy stained with combined KIM-1 immunohistochemistry and periodic acid–Schiff (PAS). The solid yellow box highlights a cortical area with KIM-1–positive proximal tubules that do not show overt tubular atrophy on routine morphology; this region was selected as the primary target for vacuolation assessment. In contrast, the dashed yellow box indicates an area containing KIM-1–positive tubules within an atrophic background, which was excluded from vacuolation scoring. (**B**) higher magnification of the solid yellow boxed area in (**A**), demonstrating KIM-1–positive proximal tubular epithelial cells with preserved tubular caliber, allowing reliable evaluation of cytoplasmic vacuolation. Immunohistochemical visualization enables discrimination of true epithelial vacuolation from processing-related artifacts that are difficult to assess on PAS staining alone. (**C**) higher magnification of the dashed yellow boxed area in (**A**), showing KIM-1–positive proximal tubules located in an atrophic region; these tubules were analyzed separately and were not included in vacuolation scoring. (**D**) further magnification of the area shown in (**C**), highlighting KIM-1–positive proximal tubular epithelial cells within atrophic tubules. (**E**) serial section from the same region stained for hypoxia-inducible factor–1α (HIF-1α), showing minimal to absent nuclear staining at one hour after reperfusion, consistent with the ultra-early time point examined in this study

A total of 51 adult living-donor kidney transplant recipients with available baseline biopsy specimens were included in the final analysis. To enable reliable assessment of ultra-early epithelial injury, we first established strict morphological criteria for the selection of proximal tubules suitable for vacuolation analysis. In one-hour kidney allograft biopsies stained with combined KIM-1 immunohistochemistry and PAS, KIM-1–positive proximal tubules were observed in both non-atrophic and atrophic cortical regions (Fig. 1A). Low-power examination demonstrated that proximal tubules located within overtly atrophic areas frequently exhibited reduced tubular caliber, distorted architecture, and background fibrosis (Fig. 1A, dashed yellow box). Although these tubules often showed strong KIM-1 immunoreactivity, their underlying chronic structural alterations precluded reliable evaluation of acute cytoplasmic vacuolation. Accordingly, KIM-1–positive tubules within atrophic regions were excluded from vacuolation scoring and analyzed separately (Fig. 1C, D).

Scale bars: 100 μm.

In contrast, KIM-1–positive proximal tubules located in morphologically preserved cortical regions without evident atrophy retained normal tubular caliber and epithelial continuity (Fig. 1A, solid yellow box; Fig. 1B). In these regions, KIM-1 immunohistochemistry enabled precise identification of injured epithelial cells and allowed discrimination of true cytoplasmic vacuolation from processing-related artifacts that are difficult to assess on PAS staining alone. These non-atrophic KIM-1–positive proximal tubules were therefore selected as the primary target for subsequent vacuolation scoring.

Serial sections stained for hypoxia-inducible factor–1α (HIF-1α) demonstrated minimal to absent nuclear staining at one hour after reperfusion (Fig. 1E), consistent with the limited activation of hypoxia-responsive pathways at this ultra-early reperfusion time point.

### Definition of a KIM-1–associated proximal tubular vacuolation score

Based on the selected target tubules, we established a semi-quantitative scoring system to grade ultra-early proximal tubular injury by integrating KIM-1 expression with cytoplasmic vacuolation (Fig. 2). This KIM-1–associated vacuolation score ranged from 0 to 3 and reflected increasing degrees of epithelial instability rather than established necrosis.

Score 0 represented proximal tubules with preserved epithelial morphology, intact cytoplasm, and absence of KIM-1 immunoreactivity or vacuolation. Score 1 was defined by focal and weak KIM-1 positivity in a limited number of epithelial cells accompanied by minimal vacuolation without architectural distortion. Score 2 indicated moderate KIM-1 expression in multiple proximal tubules with clearly identifiable cytoplasmic vacuoles partially occupying the cytoplasm while epithelial continuity was maintained. Score 3 represented strong and diffuse KIM-1 expression associated with prominent and extensive vacuolation, in some cases accompanied by focal epithelial cell detachment.

This scoring system was designed to capture a continuum of sublethal epithelial stress occurring immediately after reperfusion, emphasizing membrane remodeling and cytoplasmic instability rather than irreversible cell loss.

The clinical characteristics of the study cohort, including donor and recipient demographics, ischemia times, immunosuppressive parameters, and baseline renal function, are summarized in Table 1.

**Table 1:** Recipient, donor, transplant, histological, biomarker, and outcome characteristics

Characteristic	Overall (n = 51)
**Recipient characteristics**	
Age, years	49.82 [±13.04]
Sex, male, n (%)	21 [41.1]
Body mass index, kg/m^2^	24.39 [19.97 - 27.18]
**Donor characteristics**	
Donor age, years	59.18 [±11.37]
Donor creatinine, μmol/L	61.88 [±13.26]
Donor eGFR, mL/min/1.73 m2	78.78 [65.02 - 86.51]
**Transplant-related factors**	
HLA mismatch (total)	3 [2 - 5]
ABO incompatible, n (%)	24 [47.1]
Cold ischemia time, min	283 [152 - 345]
Warm ischemia time, min	8 [7 - 10]
**Immunosuppression**	
Pre-transplant tacrolimus trough, ng/mL	12.3[8.8 - 16.4]
**Histological and biomarker findings at 1 hour**	
Tubular vacuolation score	2 [1-2]
KIM-1–positive area (whole section), %	1.60 [0.81 - 2.30]
KIM-1–positive area (medullary ray), %	3.80 [1.72 - 5.50]
HIF-1α H-score	0.538 [0.129 - 1.403]
**Outcomes**	
Serum creatinine at 1 week, μmol/L	139.7 [107.0 - 184.8]
eGFR at 1 week, mL/min/1.73 m2	41.27 [±20.25]
eGFR at 1 month, mL/min/1.73 m2	40.94 [34.58 - 54.77]

Data are presented as mean ± standard deviation, median [interquartile range], or number (%), as appropriate. Estimated glomerular filtration rate (eGFR) was calculated using the CKD-EPI equation and is expressed as mL/min/1.73 m^2^. Body mass index (BMI) is expressed as kg/m^2^. Cold and warm ischemia times are expressed in minutes. Tubular vacuolation score was evaluated in proximal tubules within the medullary ray at 1 hour after reperfusion. KIM-1–positive area represents the percentage of immunopositive area within the whole cortical section or within the medullary ray, as indicated. HIF-1α expression was assessed using the H-score method. Pre-transplant tacrolimus trough concentration was measured immediately before transplantation. HLA, human leukocyte antigen; KIM-1, kidney injury molecule-1; HIF-1α, hypoxia-inducible factor-1 alpha; eGFR, estimated glomerular filtration rate; BMI, body mass index

### Relationship between KIM-1–associated vacuolation score, cold ischemia time, and early graft function

**Fig. 3:**
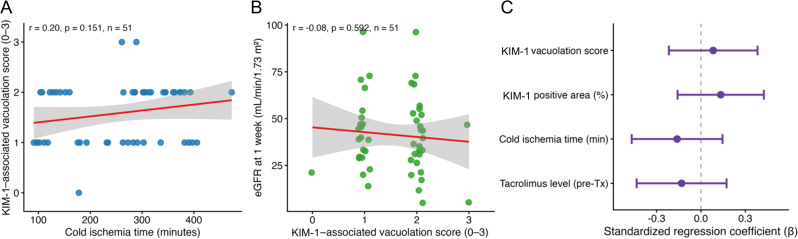
Association of KIM-1–associated vacuolation with ischemic exposure and early renal function. (**A**) scatter plot showing the relationship between cold ischemia time (CIT) and the KIM-1–associated vacuolation score assessed in one-hour post-reperfusion baseline biopsies. A fitted linear regression line with 95% confidence interval is shown. Cold ischemia time showed a weak positive trend with vacuolation score, suggesting that the score captures ischemia-related epithelial changes at the ultra-early reperfusion phase, although the association did not reach statistical significance. (**B**) relationship between the KIM-1–associated vacuolation score and estimated glomerular filtration rate (eGFR) measured one week after transplantation. Individual data points are displayed with jitter to illustrate distribution, together with a fitted linear regression line and 95% confidence interval. Higher vacuolation scores tended to be associated with lower early post-transplant renal function, although this trend did not reach statistical significance. (**C**) forest plot of standardized regression coefficients derived from multivariable linear regression analysis evaluating factors associated with early post-transplant renal function (eGFR at 1 week). The model included the KIM-1–associated vacuolation score, QuPath-derived KIM-1–positive tubular area (whole cortical ROI), cold ischemia time, and pre-transplant tacrolimus trough concentration. Points indicate standardized β coefficients and horizontal bars represent 95% confidence intervals. None of the variables showed an independent association with early renal function, indicating that KIM-1–associated vacuolation reflects ultra-early epithelial instability rather than established injury burden at this time point

We next examined the relationship between the KIM-1–associated vacuolation score and cold ischemia time (CIT). Scatter plot analysis demonstrated a weak positive trend between CIT and vacuolation score, although this association did not reach statistical significance (Fig. 3A). This finding suggests that at the one-hour reperfusion time point, early epithelial instability may not be determined by the duration of cold ischemia alone.

To assess the functional relevance of the vacuolation score, we analyzed its relationship with early post-transplant renal function. The KIM-1–associated vacuolation score showed a weak inverse association with estimated glomerular filtration rate (eGFR) at one week after transplantation (Fig. 3B), indicating that higher degrees of ultra-early epithelial instability tended to be associated with lower early graft function, although again without reaching statistical significance.

Multivariable linear regression analyses were performed to evaluate independent predictors of early graft function, with eGFR at 1 week after transplantation as the dependent variable.

A parsimonious model including the KIM-1–associated vacuolation score, QuPath-derived KIM-1–positive tubular area (whole cortical ROI), cold ischemia time, and pre-transplant tacrolimus trough concentration demonstrated no significant independent associations with eGFR at 1 week (Fig. 3C; Table 2). All standardized regression coefficients (β) were modest in magnitude, and their 95% confidence intervals crossed the null value, indicating limited explanatory power at this ultra-early time point.

**Table 2:** Multivariable linear regression analysis of factors associated with estimated glomerular filtration rate (eGFR) at 1 week after kidney transplantation

Predictor	β (95% CI)	p value
TEC vacuolation score, score 1 vs 0	5.78 (−9.56 to 21.12)	0.451
TEC vacuolation score, score 2 vs 0	4.11 (−11.18 to 19.39)	0.59
TEC vacuolation score, score 3 vs 0	−2.72 (−27.76 to 22.32)	0.827
Cold ischemia time (per min)	−0.031 (−0.097 to 0.035)	0.348
Warm ischemia time (per min)	−0.89 (−3.00 to 1.22)	0.398
Pre-transplant tacrolimus trough (per ng/mL)	−0.097 (−0.679 to 0.485)	0.737
Donor eGFR (per 1 mL/min/1.73 m^2^)	0.289 (−0.119 to 0.697)	0.16
Tubular interstitial edema by Masson trichrome staining (per 1%)	0.619 (−1.49 to 2.73)	0.556
KIM-1–positive area (Medullary ray)	1.87 (−1.45 to 5.20)	0.261
KIM-1–positive area (Whole section)	−0.85 (−4.22 to 2.52)	0.614
HIF-1α H-score at 1 h (per 1-point)	−0.50 (−3.17 to 2.18)	0.71

Regression coefficients (β) with 95% confidence intervals (CI) are shown. The outcome variable was estimated glomerular filtration rate (eGFR) at 1 week after kidney transplantation, expressed as mL/min/1.73 m^2^. Tubular epithelial cell (TEC) vacuolation in proximal tubules of the medullary ray at 1 hour after reperfusion was treated as a categorical variable, with score 0 as the reference category. Tubulointerstitial edema was quantified as the percentage of edematous area on Masson trichrome–stained sections. Cold and warm ischemia times are expressed in minutes. Pre-transplant tacrolimus trough concentration is expressed in ng/mL. Donor eGFR was calculated using the CKD-EPI equation. KIM-1–positive area and HIF-1α H-score were assessed by immunohistochemistry.

To further evaluate these findings, an expanded multivariable model incorporating additional prespecified clinical, histological, and molecular covariates was analyzed (Table 2). In this full model, vacuolation score categories (scores 1–3, reference score 0), cold and warm ischemia times, pre-transplant tacrolimus trough concentration, donor eGFR, tubulointerstitial edema assessed by Masson trichrome staining (Supplementary Figure 3), QuPath-derived KIM-1–positive area in both the medullary ray and whole cortical regions, and HIF-1α H-score at 1 hour after reperfusion were included as covariates.

None of these variables showed an independent association with eGFR at 1 week. Effect estimates for vacuolation score categories were small and directionally inconsistent, with wide confidence intervals. Similarly, ischemia times, pre-transplant tacrolimus exposure, donor renal function, histological edema, quantitative KIM-1–positive area, and HIF-1α nuclear activation all demonstrated non-significant associations with modest effect sizes. Taken together, these results suggest that, within the limits of this cohort, no single morphologic, molecular, or clinical parameter independently explained early graft function at one week in the context of one-hour reperfusion biopsies, consistent with the limited sample size and the ultra-early phase of injury assessed in this study.

### Morpho-molecular dissociation between KIM-1–associated epithelial instability and hypoxia-driven transcriptional responses

**Fig. 4:**
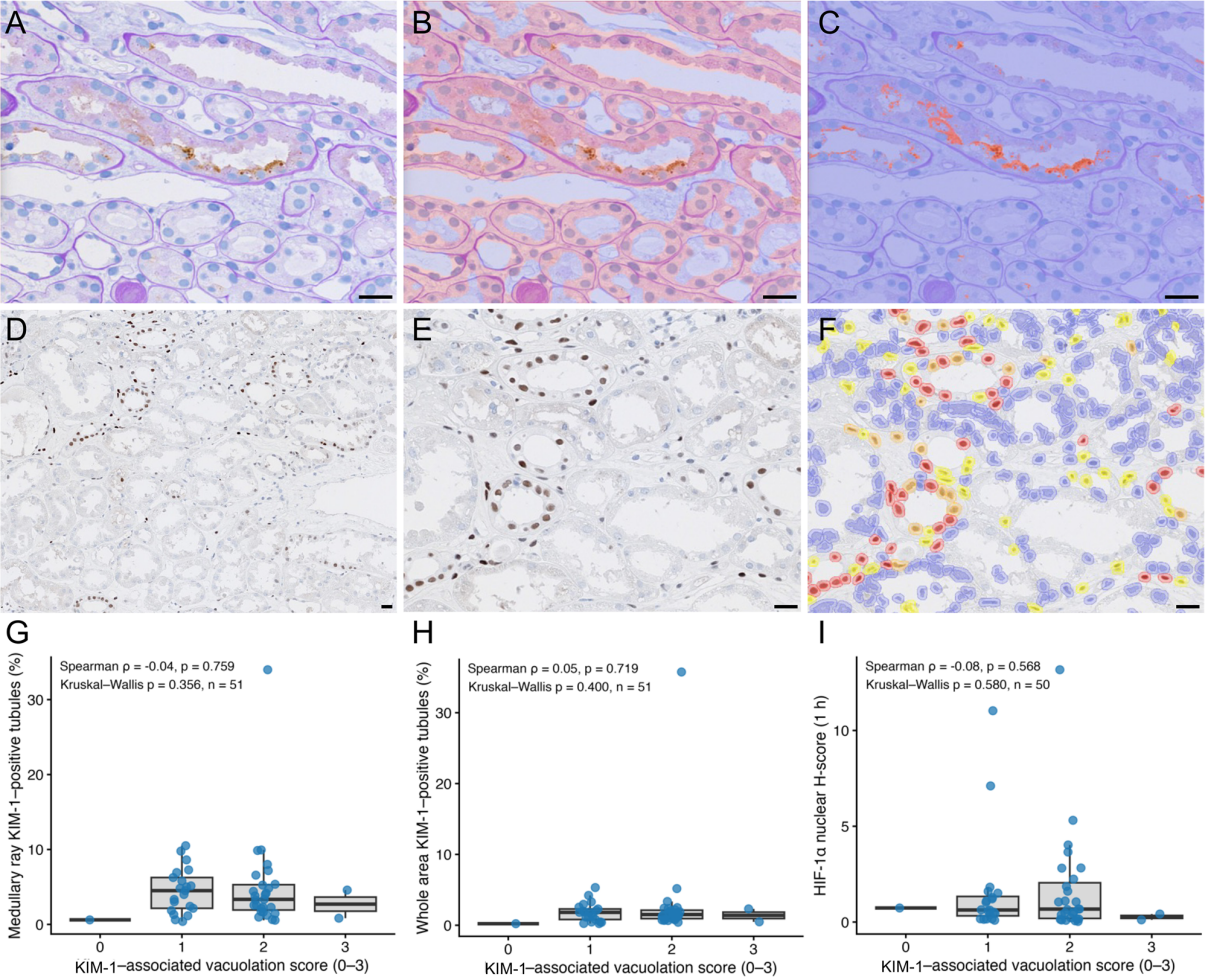
KIM-1–associated vacuolation score is independent of KIM-1–positive area and HIF-1α activation at one hour after reperfusion. (**A**–**C**) representative images of KIM-1 immunohistochemistry and corresponding QuPath-based detection of KIM-1–positive proximal tubular profiles in morphologically non-atrophic cortical regions. QuPath-based analysis accurately identifies KIM-1–positive tubules, enabling quantitative assessment of KIM-1–positive tubular area across the entire analyzed cortical region as well as within medullary ray–restricted regions. (**D**–**F**) representative images of hypoxia-inducible factor–1α (HIF-1α) immunohistochemistry and corresponding QuPath-based nuclear detection. HIF-1α–positive nuclei are sparsely detected at one hour after reperfusion, indicating minimal activation of hypoxia-driven transcriptional responses at this ultra-early time point. (**G**–**H**) relationship between the KIM-1–associated vacuolation score (0–3) and the percentage of KIM-1–positive tubular area, calculated as the proportion of total tubular epithelial area classified as KIM-1–positive by QuPath, within the medullary ray region (**G**) and across the whole analyzed cortical area (**H**). No significant association was observed, indicating that the vacuolation score does not simply reflect the extent of KIM-1–positive tubular area. (**I**) relationship between the KIM-1–associated vacuolation score and HIF-1α nuclear H-score at 1 hour after reperfusion. The HIF-1α H-score was calculated using a QuPath-based simplified algorithm integrating the proportion of positive nuclei and their mean staining intensity. No significant association was observed, supporting a dissociation between early epithelial structural instability and hypoxia-driven nuclear signaling at this time point. *n* = 51 for panels (**G**–**H**) and *n* = 50 for panel (**I**), with one case excluded from HIF-1α analysis due to insufficient immunohistochemical staining quality. Scale bars: 20 μm

KIM-1–positive tubular area, assessed both in the medullary ray region and across the whole cortical area, showed no significant correlation with the KIM-1–associated vacuolation score (Fig. 4H, I). These findings indicate that the vacuolation score reflects qualitative epithelial instability rather than simply the extent of KIM-1–positive injury burden.

Consistent with the findings in Fig. 1, quantitative analysis of HIF-1α nuclear staining demonstrated low levels of activation at one hour after reperfusion, with no significant association between HIF-1α H-score and the vacuolation score (Fig. 4J). These quantitative QuPath analyses supported the interpretation that the KIM-1–associated vacuolation score is not simply explained by either the extent of KIM-1–positive tubular area or HIF-1α nuclear activation at one hour after reperfusion (Fig. 4). This lack of correlation is consistent with the interpretation that hypoxia-driven transcriptional programs are not yet fully engaged at this ultra-early time point and may be uncoupled from the early morphological changes assessed by KIM-1–associated vacuolation. To further assess whether these ultra-early morphological features were associated with renal function at other early time points, additional multivariable regression analyses were performed using alternative functional outcomes. Consistent with the primary analyses, no independent associations were observed between the KIM-1–associated vacuolation score, ischemia-related parameters, KIM-1–positive area, or HIF-1α H-score and renal function, with modest effect sizes and wide confidence intervals (Supplementary Table 1).

## Discussion

In the present study, we explored whether a proof-of-concept approach combining KIM-1 immunoreactivity and proximal tubular morphology in one-hour kidney allograft biopsies could help characterize ultra-early epithelial stress responses.

Consistent with prior experimental and human data, nuclear accumulation of hypoxia-inducible factor–1α (HIF-1α) was minimal at this early reperfusion time point, indicating that classical hypoxia-responsive gene programs are not yet fully engaged one hour after reperfusion [14–16]. This temporal dissociation provides an essential biological framework for interpreting the present findings.

Renal ischemia–reperfusion injury is a dynamic, time-dependent process in which early membrane and cytoskeletal perturbations precede overt transcriptional reprogramming. Experimental studies have shown that ischemia initially disrupts actin organization, vesicular trafficking, and epithelial polarity in proximal tubular cells, resulting in cytoplasmic vacuolation and membrane instability well before cell death or fibrotic signaling becomes apparent [6, 17]. In this context, the KIM-1–associated vacuolation score defined in our study should not be interpreted as a measure of cumulative injury burden, but rather as an index of early epithelial instability reflecting sublethal stress at the level of the plasma membrane and intracellular vesicle system. Importantly, the KIM-1–associated vacuolation score did not correlate with the overall extent of KIM-1–positive tubular area, indicating that it does not simply reflect the quantity of KIM-1–expressing epithelium. The absence of a monotonic relationship between vacuolation severity and KIM-1–positive area suggests that the score captures qualitative epithelial instability within KIM-1–positive tubules. This interpretation is further supported by the lack of association with HIF-1α nuclear activation, underscoring that the observed vacuolar changes represent an ultra-early epithelial stress state that precedes hypoxia-driven transcriptional responses.

KIM-1 is uniquely suited to anchor such an assessment. Although the degree of KIM-1 positivity was limited at one hour after reperfusion, prior experimental studies have demonstrated that KIM-1 can be induced rapidly in stressed but viable proximal tubular epithelial cells during the earliest phases of acute injury [18, 19]. Nevertheless, because zero-time biopsies from non-ischemic kidneys were not available in the present study, we cannot exclude the possibility that low-level baseline KIM-1 expression may also occur in minimally injured kidneys. By combining KIM-1 immunohistochemistry with PAS morphology, we aimed to selectively identify non-atrophic proximal tubules undergoing ultra-early stress and to distinguish true epithelial vacuolation from processing-related artifacts. This KIM-1–guided approach may improve the specificity of vacuolation assessment by focusing on tubules with molecular evidence of epithelial injury, rather than relying on morphology alone. This methodological approach is particularly important in the transplant setting, where baseline chronic changes and fixation-related artifacts often complicate morphological interpretation.

Spatially, our analysis focused on proximal tubules within medullary ray regions, which are known to be especially vulnerable to ischemic stress due to their high metabolic demand and relatively low oxygen tension [20, 21]. The preferential involvement of these regions supports the biological plausibility of our scoring system and aligns with established concepts of outer medullary susceptibility in ischemic acute kidney injury. Importantly, vacuolation severity showed a tendency to associate with cold ischemia time, further reinforcing the interpretation that the observed morphological changes may reflect early ischemia-related epithelial stress rather than post-reperfusion inflammatory or toxic effects.

Despite clear morphological evidence of early epithelial stress, neither serum creatinine nor estimated glomerular filtration rate (eGFR) at one week showed strong correlations with the vacuolation score. This apparent dissociation should not be viewed as a limitation of the morphological assessment but rather as a reflection of the well-recognized insensitivity of conventional functional markers to detect early or subclinical tubular injury [22, 23]. Indeed, functional parameters integrate whole-organ filtration and often remain preserved until substantial nephron loss or hemodynamic alterations occur. Our findings therefore highlight the complementary value of tissue-based biomarkers for capturing injury states that are invisible to routine clinical measurements.

We also considered potential confounding effects of calcineurin inhibitor exposure, as tubular vacuolation has historically been associated with calcineurin inhibitor toxicity. However, classical calcineurin inhibitor–related vacuolation is typically observed after sustained exposure and is accompanied by distinct arteriolar and interstitial changes [24]. Moreover, early indication biopsies have shown that morphological alterations at ultra-early time points often reflect ischemic and donor-related factors rather than immunosuppressive toxicity [25]. The lack of a strong association between tacrolimus levels and vacuolation severity in our cohort further supports the interpretation that the observed changes primarily represent ischemia-related epithelial stress.

From a broader perspective, early epithelial instability may represent a biologically relevant vulnerability state that influences long-term graft trajectories. Experimental and clinical studies have increasingly emphasized that incomplete or maladaptive recovery from early tubular stress can predispose to chronic injury, even in the absence of overt acute rejection or early functional decline [26, 27]. Within this framework, the KIM-1–associated vacuolation score may help characterize subtle epithelial injury pathways that could influence later graft recovery, although this possibility requires validation in larger cohorts.

Several limitations warrant consideration. Because several associations did not reach statistical significance, the present findings should be interpreted as exploratory and hypothesis-generating rather than definitive. In addition, we did not establish whether vacuolation assessed without reference to KIM-1 immunoreactivity would show similar associations with cold ischemia time or early renal function. Future studies should directly compare KIM-1–guided scoring with morphology-only assessment to determine the incremental value of KIM-1 immunostaining. The sample size was modest, limiting statistical power to detect small effect sizes and precluding definitive conclusions regarding long-term outcomes. Furthermore, as this was a single-center study restricted to living-donor kidney transplantation, the generalizability of these findings to other transplant populations, particularly deceased-donor cohorts and different preservation or immunosuppressive settings, remains to be determined. Because living-donor transplantation is generally associated with shorter ischemia times and less severe baseline injury than deceased-donor transplantation, the relatively limited degree of injury in this cohort may also have reduced the discriminatory power of the proposed scoring approach. Future validation in deceased-donor cohorts with broader injury severity distributions will therefore be important. In addition, the retrospective nature of this study introduces the possibility of selection bias and unmeasured confounding. Although we included consecutive eligible cases and performed histological evaluations in a blinded fashion, residual bias related to patient selection, perioperative management, or unrecorded donor and recipient characteristics cannot be fully excluded. Nevertheless, the consistency of these trends with established biological principles supports the plausibility of our interpretation. Importantly, the aim of this study was not to establish an immediate clinical decision tool, but rather to explore a potential morpho-molecular readout of ultra-early epithelial injury.

In conclusion, combined assessment of KIM-1 expression and proximal tubular vacuolation in one-hour reperfusion biopsies captured morphologic features of ultra-early epithelial stress occurring before overt HIF-1α activation. Although no significant associations with early graft function were identified, this proof-of-concept approach may provide a framework for future studies investigating early epithelial stress responses following kidney transplantation.

## Electronic supplementary material

Below is the link to the electronic supplementary material.

Supplementary material 1

Supplementary material 2

## Data Availability

The individual-level data supporting the findings of this study are provided in the Supporting Information (Supporting Table S1).

## Abbreviations

AKI: Acute kidney injury
BMI: Body mass index
CIT: Cold ischemia time
Cr: Creatinine
DAB: 3,3′-diaminobenzidine
eGFR: Estimated glomerular filtration rate
HIF-1α: Hypoxia-inducible factor-1 alpha
HLA: Human leukocyte antigen
IRI: Ischemia–reperfusion injury
KIM-1: Kidney injury molecule-1; PAS, periodic acid–Schiff
ROI: Region of interest
WIT: Warm ischemia time
