# Supplementary material for: Combined morphologic and immunohistochemical assessment of KIM-1-positive proximal tubular vacuolation in one-hour kidney allograft biopsies
Source: BMC Nephrol. 2026 Jun 5;27:469. doi: 10.1186/s12882-026-05088-5 (PMC13463605; doi:10.1186/s12882-026-05088-5)
Supplement: Supplementary file 1 [file 12882_2026_5088_MOESM1_ESM.pdf]

Supplementary File for

A morpho-molecular indicator of ultra-early epithelial instability based on KIM-1 expression and proximal tubular vacuolation in one-hour kidney allograft biopsies

Keishu Kawanishi[1,2], Mayu Shimokawa[1,2], Dedong Kang[1], Masaki Baba[1,3], Yojiro Kato[4], Osamu Yoshitake[4], Fumihiko Koiwa[2], Kazuho Honda[1,5], and Kunio Kawanishi[1,6]*

Affiliations:

[1]Department of Anatomy, Showa Medical University School of Medicine, Hatanodai, Shinagawa-ku, Tokyo, Japan

[2]Internal Medicine (Nephrology), Showa Medical University Fujigaoka Hospital, Yokohama, Japan

[3]Department of Diagnostic Pathology, Institute of Medicine, University of Tsukuba, Ibaraki, Japan,

[4]Kidney Transplant Center, Showa Medical University Hospital

[5]Department of Pathology and Laboratory Medicine, Showa Medical University School of Medicine, Hatanodai, Shinagawa-ku, Tokyo, Japan

[6]Department of Experimental Pathology, Institute of Medicine, University of Tsukuba, Ibaraki, Japan,

*Correspondence: Kunio Kawanishi (kukawanishi@med.showa-u.ac.jp), Department of Anatomy, Showa Medical University School of Medicine, 1-5-8 Hatanodai, Shinagawa-ku, Tokyo, Japan, 142-8555

This PDF file contains Supplementary Methods, Supplementary Figures 1–3 and Supplementary Table 1.

Supplementary Methods

Quantification of KIM-1–positive tubular epithelial cells

KIM-1–positive tubular epithelial cells were quantified using QuPath (version 0.6.0-rc3). From whole-slide images, only the renal cortex was selected for analysis, and medullary regions were excluded. Within the cortical region, glomeruli and chronically atrophic tubules were manually excluded using QuPath to avoid confounding by pre-existing structural alterations.KIM-1–positive areas were subsequently identified within the remaining analysis region using a classifier-based approach, and the proportion of KIM-1–positive area was calculated relative to the total analyzed cortical area.

Quantification of interstitial edema

Interstitial edema was evaluated on Masson's trichrome–stained sections. Digital whole-slide images were analyzed using QuPath. Interstitial edema was distinguished from tubulointerstitial fibrosis based on established staining characteristics and morphological features, including pale-staining expanded interstitial spaces lacking dense collagen deposition. Areas corresponding to tubular lumina, glomeruli, and vascular structures were excluded from the analysis. The extent of interstitial edema was quantified as the area of edema divided by the total interstitial area within the defined region of interest and expressed as a percentage.

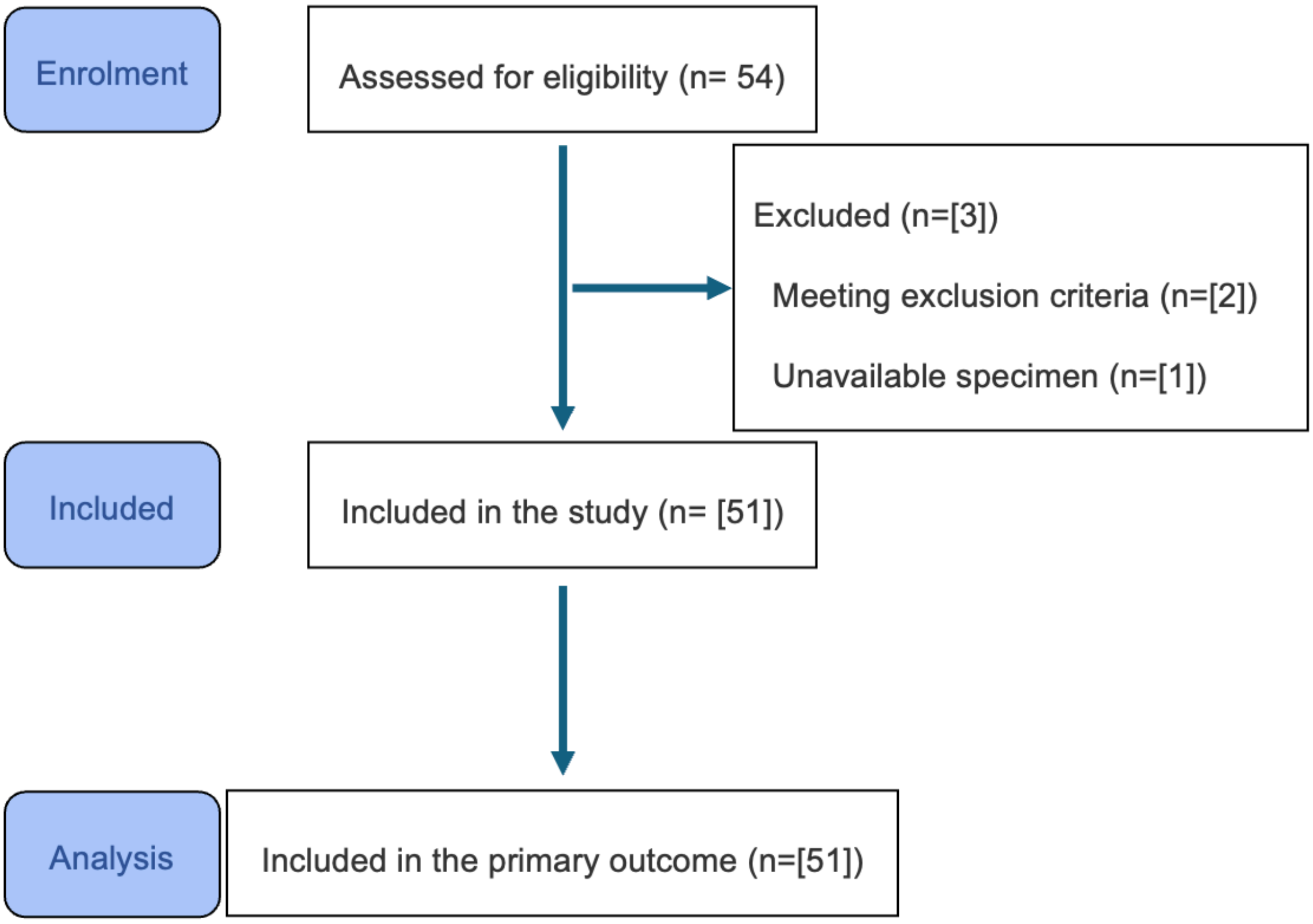

Supplementary Figure 1. Flow diagram of patient inclusion and analysis in this observational study.

Flow diagram illustrating the assessment of eligibility, exclusions, and final inclusion of kidney transplant recipients in the primary analysis. A total of 54 patients were assessed for eligibility. Three patients were excluded, including two who met exclusion criteria and one with unavailable biopsy specimens. Consequently, 51 patients were included in the study and analyzed for the primary outcome. The diagram is presented in a CONSORT-style format for clarity, although this study represents a retrospective observational cohort.

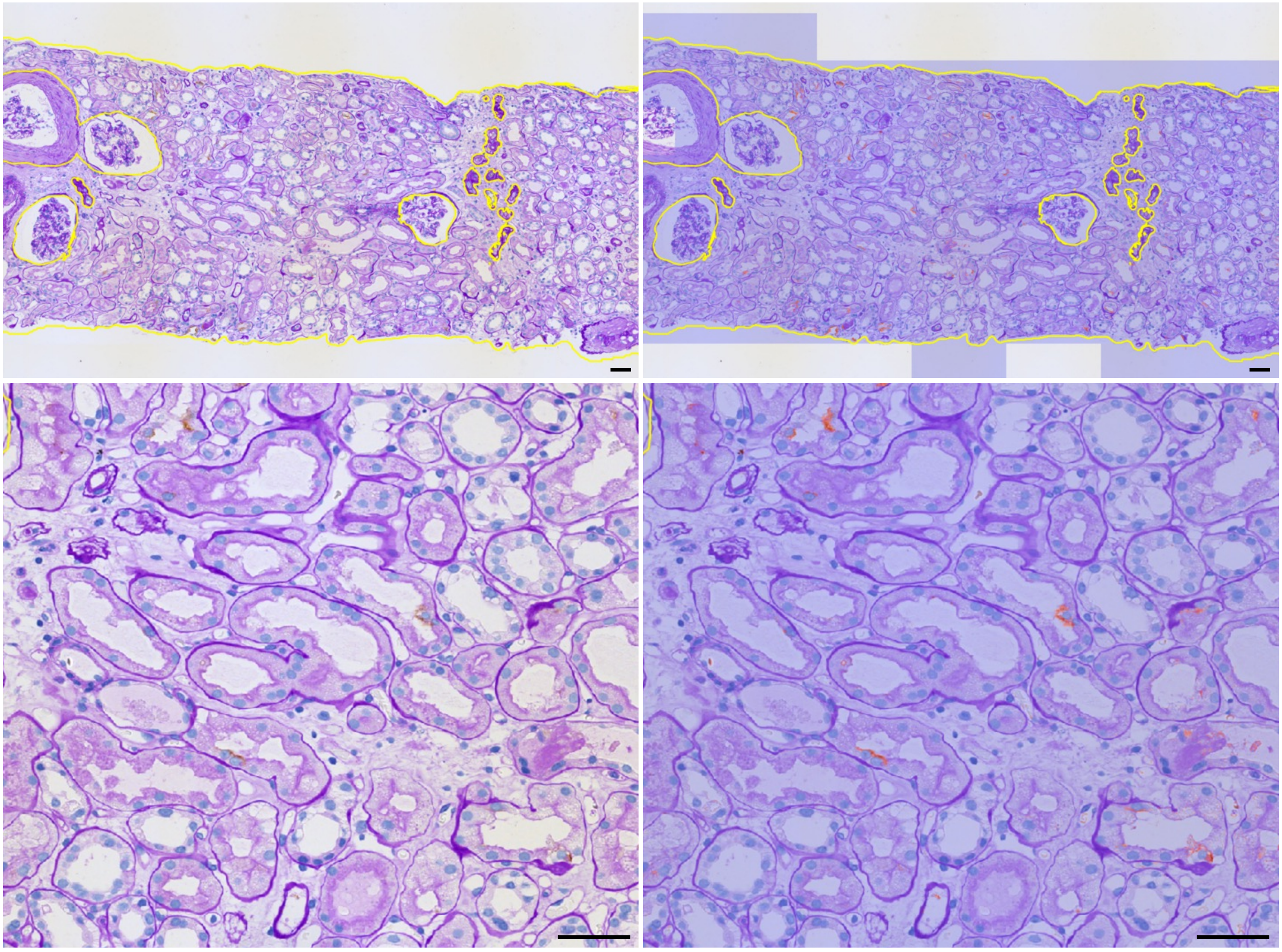

Supplementary Figure 2. Region of interest (ROI) definition and QuPath-based detection of KIM-1–positive tubular area

(A) Representative whole-slide image illustrating the region of interest (ROI) used for quantification of the KIM-1–positive area. The analysis was restricted to the renal cortex, while medullary regions, glomeruli, and chronically atrophic tubules were manually excluded. (B) Detection of KIM-1–positive tubular epithelial cells within the defined ROI using a QuPath-based classifier, identifying diaminobenzidine (DAB)–positive signals corresponding to KIM-1 immunoreactivity. (C) Higher-magnification view of the cortical tubular area included in the ROI, highlighting proximal tubules subjected to quantitative analysis. (D) Corresponding high-magnification image demonstrating accurate classifier-based detection of KIM-1–positive tubular epithelial cells. Scale bars represent 50 μm.

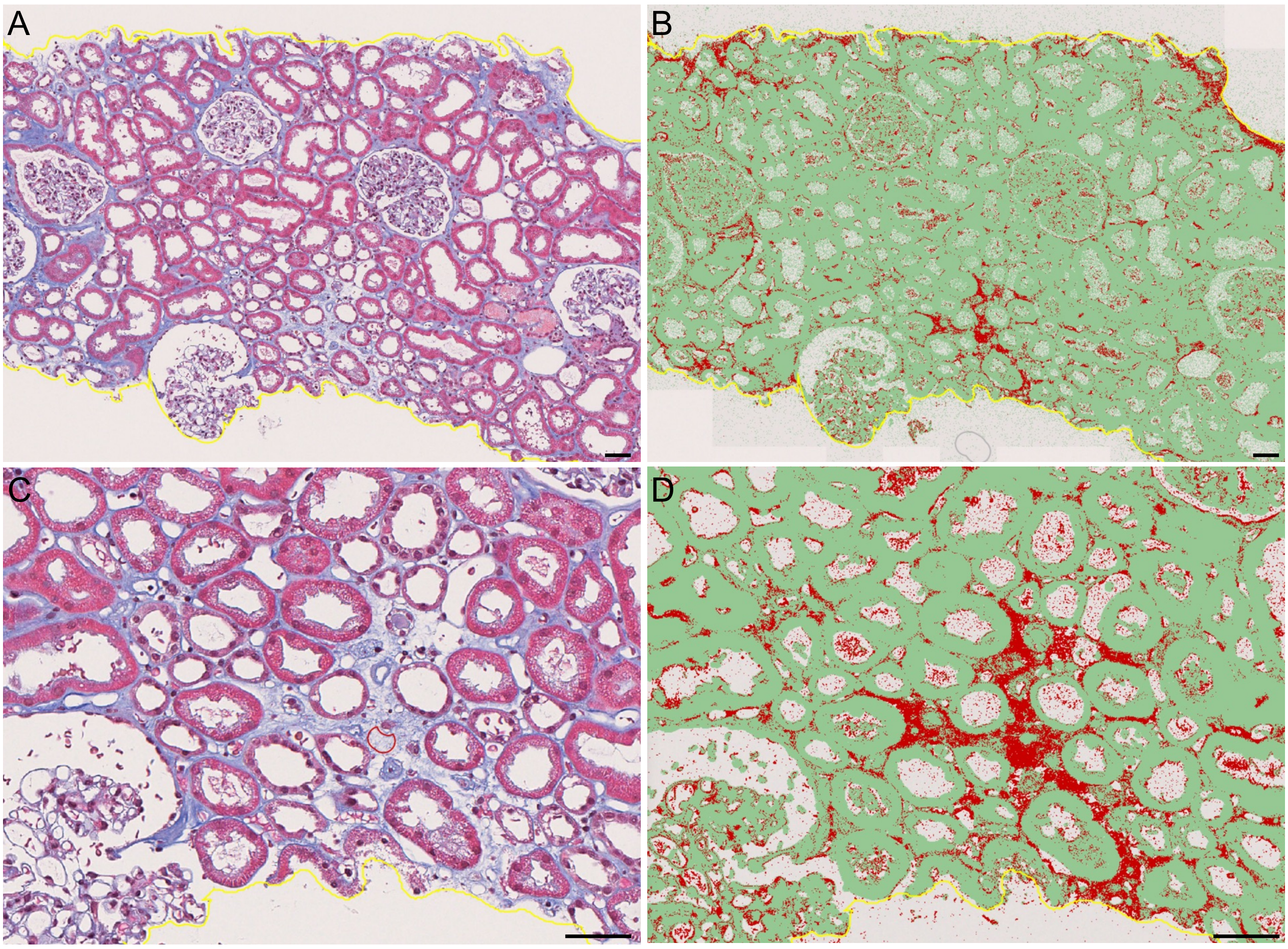

Supplementary Figure 3. QuPath-based assessment of interstitial edema on Masson's trichrome–stained sections

(A) Representative whole-slide image of a Masson's trichrome–stained renal biopsy section showing the region of interest (ROI) used for interstitial edema assessment.

(B) QuPath-based segmentation of the interstitial compartment within the ROI, with areas classified as interstitial edema highlighted. (C) Higher-magnification view of the cortical tubulointerstitium illustrating morphologic features of interstitial edema on Masson's trichrome staining. (D) Corresponding high-magnification image demonstrating QuPath-based detection and quantification of interstitial edema, distinguished from fibrotic areas by staining intensity and tissue architecture. Scale bars represent 50 μm.

Supplementary Table 1. Multivariable linear regression analysis of factors associated with estimated glomerular filtration rate (eGFR) at 1 month after kidney transplantation

Predictor	β (95% CI)	P value
TEC vacuolation score, score 1 vs 0	-0.70 (-12.56 to 11.15)	0.905
TEC vacuolation score, score 2 vs 0	2.97 (-8.84 to 14.79)	0.613
TEC vacuolation score, score 3 vs 0	-2.10 (-21.45 to 17.25)	0.827
Cold ischemia time (per min)	-0.028 (-0.079 to 0.023)	0.278
Warm ischemia time (per min)	-0.27 (-1.90 to 1.36)	0.742
Pre-transplant tacrolimus trough (per ng/mL)	-0.205 (-0.655 to 0.245)	0.363
Donor eGFR (per 1 mL/min/1.73 m²)	0.236 (-0.079 to 0.552)	0.138
Tubular interstitial edema by Masson trichrome staining (per 1%)	-0.580 (-2.21 to 1.05)	0.477
KIM-1–positive area (Medullary ray)	1.26 (-1.30 to 3.83)	0.325
KIM-1–positive area (Whole section)	-0.89 (-3.50 to 1.71)	0.492
HIF-1α H-score at 1 h (per 1-point)	-1.02 (-3.08 to 1.05)	0.324

Regression coefficients (β) with 95% confidence intervals (CI) are shown. The outcome variable was estimated glomerular filtration rate (eGFR) at 1 month after kidney transplantation, expressed as mL/min/1.73 m². Tubular epithelial cell (TEC) vacuolation in proximal tubules of the medullary ray at 1 hour after reperfusion was treated as a categorical variable, with score 0 as the reference category. Tubulointerstitial edema was quantified as the percentage of edematous area on Masson trichrome–stained sections. Cold and warm ischemia times are expressed in minutes. Pre-transplant tacrolimus trough concentration is expressed in ng/mL. Donor eGFR was calculated using the CKD-EPI equation. KIM-1–positive area and HIF-1α H-score were assessed by immunohistochemistry.
